# More than Just Child’s Play?: An Experimental Investigation of the Impact of an Appearance-Focused Internet Game on Body Image and Career Aspirations of Young Girls

**DOI:** 10.1007/s10964-017-0659-7

**Published:** 2017-03-18

**Authors:** Amy Slater, Emma Halliwell, Hannah Jarman, Emma Gaskin

**Affiliations:** Centre for Appearance Research, University of the West of England, Bristol, UK

**Keywords:** Internet games, Body image, Career aspirations, Sexualization, Appearance, Girls

## Abstract

In recent years, elements of the modern environment (such as television, Internet, toys and clothes) have been criticized for having an increasingly sexualized or appearance focus, which has been suggested to be detrimental to girls’ development. The current study examined the impact of an appearance-focused Internet game on young girls’ body image and career cognitions and aspirations. Eighty British girls aged 8–9 years were randomly assigned to play an appearance-focused or a non-appearance focused game for 10 minutes. Girls in the appearance-focused game condition displayed greater body dissatisfaction compared to the control condition. Type of game did not impact girls’ perceived capacity to do various jobs. However, girls who played the appearance-focused game reported a greater preference for feminine careers compared to the control group. This provides preliminary evidence that appearance-focused Internet games may be detrimental to young girls’ body image and aspirations. Internet games should be included in our consideration of influential messages for young girls.

## Introduction

In recent years, public debate has intensified about the early sexualization of girls, or girls “growing up too quickly”. In particular, elements of the modern environment (such as magazines, television, Internet, toys, and clothes) have been suggested to be increasingly grown up and often have an appearance or sexualized focus. This concern has been reported in the American Psychological Association’s *Report of the APA Task Force on the Sexualization of Girls* (2007) as well as by the UK Government Report *Letting Children be Children* (Bailey 2011). Both reports outline that girls are growing up in a cultural environment that overly emphasizes the importance of appearance and looking “sexy” and that this may be detrimental to girls’ self-image and healthy development. A relevant and related concept is *self-objectification* (a term used interchangeably with sexualization in the APA report), which refers to the process by which girls internalize an observer’s perspective on their bodies and learn to treat themselves as objects to be valued primarily on their appearance. A sizeable body of research has demonstrated that self-objectification is associated with a host of negative consequences in adolescent girls and women, including disordered eating (e.g., Calogero et al. 2005; Greenleaf 2005; Slater and Tiggemann 2010; Tiggemann and Slater 2001; Tylka and Hill 2004), sexual dysfunction (Calogero and Thompson 2009; Steer and Tiggemann 2008), attitudes toward cosmetic surgery (Calogero et al. 2010), and depression (Muehlenkamp and Saris–Baglama 2002; Muehlenkamp et al. 2005; Tiggemann and Kuring 2004). Given these known negative consequences, it is critical for research to investigate the impact of a sexualized culture on young girls. Although environmental factors associated with objectification, or sexualization, of women and adolescent girls have been widely documented, as yet there has been limited research that has examined the issue in young girls.

The current study begins to address this gap in the literature by exploring the impact of Internet games on young girls’ body image and career aspirations. Despite being a common form of entertainment for young children, Internet games have not received any empirical investigation as to their potential impact. Here we investigate the impact of playing an Internet game that has an “appearance-focus”—the female character changes her outward physical appearance (clothes and hairstyle) in order to be appealing to a male character. The impact of brief exposure to this game on girls’ body dissatisfaction, self-objectification, and career aspirations and preferences is investigated.

### The Development of Body Image in Childhood

Children are being exposed to appearance-focused messages and sexualized portrayals at an early stage of their socio-emotional development. This is concerning as even very young children may be influenced by this exposure. From as young as 3 years old, children are able to verbally describe themselves. Over the following years, children begin to compare themselves to others, although this is initially only in relation to one person at a time (Smolak 2012). Also from the age of 3, children in Western cultures start to develop and display stereotypical beliefs that beauty (and thinness) is associated with positive characteristics (Harriger et al. 2010; Spiel et al. 2012). Around this age, young children frequently engage in pretend play (often adopting the role of a character) but may not be cognitively capable of making comparisons. However, as a child reaches 6 or 7 years old she places a greater emphasis on self-evaluation, and increases in engagement in social comparisons are apparent. Therefore, from this age it is likely that internalization of the thin ideal leads to appearance-related social comparisons, that may increase body image concerns (Anschutz et al. 2012).

According to the sociocultural theory of body image (Thompson et al. 1999), children are particularly susceptible to messages from their parents, their peers, and the media, and importantly, may lack the cognitive skills and abilities to critically evaluate messages from these sources (Kunkel et al. 2004). Both parents and peers may influence body image through direct or indirect communication and modelling (Dohnt and Tiggemann 2005; Hart et al. 2015; Jones and Crawford 2006). Notwithstanding the important role of parents and peers, the mass media are arguably the most powerful transmitter of sexualized and idealized images of both men and women (American Psychological Association Task Force on the Sexualization of Girls 2007). The mass media consistently depicts and promotes images of the thin ideal—including tall, extremely thin, moderately breasted women—that are unrealistic and unattainable for the large majority. These idealized images are not only found in adult media but are also found to be increasingly common in children’s media. For example, a content analysis of popular children’s films (e.g., *Cinderella*, *The Little Mermaid*) suggested that many films contain 10 or more body image-related messages (Herbozo et al. 2004). Understanding the impact of exposure to such messages is critical for researchers striving to both understand, and ultimately ameliorate, any negative impacts of children’s media.

### The Impact of Exposure to Media Influences

A number of meta-analyses have reported that media exposure adversely affects women and girls’ body image, self-esteem, eating behavior, and emotional well-being, and that this effect appears stronger for girls under the age of 19 years (Grabe et al. 2008; Groesz et al. 2002; Want 2009). However, despite the fact that children are likely to be particularly vulnerable to media messages as they develop attitudes, values, and beliefs (Berry 2003), very few studies have focused on the impact of media exposure on young girls. Correlational research indicates that media exposure, through magazine and television programs, is related to internalization of the thin ideal, body dissatisfaction, and dieting awareness in girls as young as 6 years old (Dohnt and Tiggemann 2006; Harrison and Hefner 2006), and more recently that exposure to television and magazines containing sexualized content is related to a preference for sexualized clothing in 6–9 year old girls (Slater and Tiggemann 2016). These associations are perhaps unsurprising considering the high levels of gender-role stereotypes and the emphasis on appearance of women in the media, who are often presented as thin, attractive, and provocatively dressed (Downs and Smith 2010; Murnen et al. 2016; Rudy et al. 2011).

In an experimental study, Hayes and Tantleff-Dunn (2010) investigated the impact of watching appearance-focused movie clips on the body image of 3–6 year old girls. Girls in the experimental condition were exposed to film clips that contained appearance-related messages (e.g., *Beauty and the Beast*), whereas the control condition were exposed to films clips with no appearance-related messages (e.g., *Dora the Explorer*). Perhaps surprisingly, results indicated that exposure to the appearance-focused clips did *not* negatively impact on girls’ body dissatisfaction, with the authors suggesting that very young children may adopt the persona of attractive characters with whom they identify rather than comparing themselves directly to the characters as is thought to be the case with slightly older children. Other experimental research has focused on the impact of sexualized toys. Dittmar et al. (2006) showed 5–8 year old girls visual images of Barbie (an unrealistically thin doll), Emme (a realistically sized doll) or no doll (control condition). Lower body esteem and the desire for a thinner body were reported among girls exposed to the Barbie condition. A recent study expanded upon this initial work and found that 5–8 year old girls who interacted with an actual Barbie doll (rather than images of Barbie) had higher thin-ideal internalization than girls who played with a non-human toy, although no impact was found on body esteem or body dissatisfaction (Rice et al. 2016). Collectively, some of the limited evidence to date appears to suggest that media-promoted ideals, through a variety of means including magazines, television, and dolls, are associated with detrimental effects on young girls’ body image.

### Gender Roles and Stereotypes

Importantly, children’s media not only depict body ideals, they also embed gender roles and stereotypes. Children learn culturally defined norms of gendered behavior through societal cues, that often result in gender stereotypes (Martin et al. 2002). According to cognitive social learning theory (Bussey and Bandura 1999), children use gender as a categorizing framework. Stereotypical gender beliefs can emerge when they are transmitted in one’s society and culture. This is particularly concerning given that evidence suggests women are under-represented in current media and, when they are present, are often portrayed in a negative manner (Rudy et al. 2011), depicted in stereotypical roles and are often sexualized through provocative clothing or nudity (Ward 2016). These findings have been mirrored in a recent content analysis of children’s products. Murnen and colleagues (Murnen et al. 2016) examined a range of children’s products in the U.S. including Halloween costumes, dolls and action figures, and Valentines cards. They found that female characters were far more likely than male characters to portray feminine-stereotyped characteristics e.g., decorative clothing and friendly facial expressions, whereas male characters were more likely to depict masculine-stereotyped characteristics e.g., functional clothing and displaying the body in motion. Therefore, it is evident that gender-stereotyped characters are portrayed in popular children’s culture and products.

Coy (2009) argued that early exposure to sexualized content may also limit young girls’ aspirations and achievements by highlighting beauty as of the upmost importance. Indeed, the cultural promotion of exaggerated gender-stereotypes may affect not only the gender-role development of a child, but also their aspirations and expectations. Tzampazi and colleagues (Tzampazi et al. 2013) used drawings and verbal justification to explore occupational preferences among Greek children and revealed that stereotypical representations of gender (e.g., girls have long hair and wear dresses) were more frequent among girls than boys, and that boys were more likely to choose careers that were “aligned” with their gender. Recent research by Sherman and Zurbriggen (2014) argued that sexualized toys may limit the career aspirations of young girls. In their experimental study, American girls aged 4–7 years were randomly allocated to play with either a Barbie or Mrs. Potato Head doll. After 5 min of exposure, the girls were asked whether they could “do” a number of occupations in the future, and whether boys could “do” these same occupations. Girls reported that boys could do significantly more occupations than they could themselves, particularly masculine-dominated ones. Furthermore, girls who played with Barbie perceived they could perform fewer occupations than boys, compared with girls who played with Mrs Potato Head. Apart from these findings, the impact of appearance-focused and sexualized messages and gender roles on career aspirations is largely under-researched.

### The Role of Internet Games

Previously, research on media exposure has been limited in its focus to traditional media formats. For example, Grabe and colleagues' (2008) meta-analysis only compiled studies that examined the impact of television programs and magazines. However, the Internet is now a powerful sociocultural influence in many children’s lives. A large-scale study of over 25,000 children across 25 European countries suggested that one third of 9–10 year olds access the Internet daily (Livingstone et al. 2011). In the UK, hours spent using the Internet have risen from 9.2 h per week in 2013 to 10.5 h per week in 2014 among 8–11 year olds (Ofcom 2014).

Between the ages of 8 and 11 years children use the Internet primarily for entertainment, including playing games. Rideout et al. (2010) surveyed more than 2000 American children and showed that games were the most commonly used computer activity among 8–10 year olds. Internet games have the potential to be more influential than alternative media due to their interactive and engaging nature, and beliefs and values including gender role identity may be perpetuated through gameplay (Oliver and Green 2001). Further, Internet games are likely another avenue through which idealized bodies and stereotypical gender roles are promoted. A content analysis of popular video console games revealed that, in comparison to male characters, female characters were underrepresented, more likely to have unrealistic body shapes, display more skin, and wear more sexualized clothing (Downs and Smith 2010). Furthermore, games aimed at children have been shown to feature thinner characters than games aimed at adults (Martins et al. 2009).

Research suggests that girls often identify with characters in games and tend to mimic them as a way of learning (Gorriz and Medina 2000). Subrahmanyam and Greenfield (1998) analyzed what appealed to girls when playing online games, and reported that they prefer role-playing games that represent real-life situations. More recently, Ofcom (2014) reported that children prefer free, multi-game websites that contain a large number of “mini games” such as *Friv*. *Friv* has been ranked as the third most popular online games website worldwide (SimilarWeb 2016), with an estimated reach of 426,000 Americans per month (Quantcast 2016).

Cross-sectional research suggests that electronic media use, including the Internet and gaming, is related to poorer psychological well-being among Northern Irish girls and lower health-related quality of life among Australian adolescents (Devine and Lloyd 2012; Mathers et al. 2009). A small number of studies have investigated the impact of Internet exposure on body image and have found associations between time spent on the Internet and poorer body image in adolescent girls (Tiggemann and Miller 2010; Tiggemann and Slater 2013) as well as in 10–12 year old girls (Tiggemann and Slater 2014). In one experimental study, Barlett and Harris (2008) found that brief exposure to a video game that featured characters with idealized bodies lowered body esteem in both men and women. However, research has yet to explore the impact of Internet games on younger children.

## The Current Study

Children’s Internet games may depict both unrealistic appearance ideals and strict gender roles. If children internalize these messages, body image disturbance may result. Further, children could take on attitudes and beliefs about gender roles from these games (gender role socialization), that could result in lowered aspirations and expectations. Previous research has largely focused on the impact of consuming traditional media images and engaging in doll play. However, Internet game play is likely to encompass important elements of both of these. Not only do Internet games contain numerous images, they are also likely to encourage some sort of character identification or adoption that may be influential. This medium provides a powerful and fertile environment for empirical research.

The current study aimed to examine the influence of an appearance-focused Internet game on the body image and career aspirations of 8–9 year old girls. It was hypothesized that girls who played an appearance-focused game would report increased body dissatisfaction and self-objectification compared to girls who played a control game. In addition, following Sherman and Zurbriggen (2014), it was also hypothesized that exposure to an appearance-focused game would result in reduced career cognitions as well as an increased preference for traditionally feminine jobs, comparative to controls.

## Method

### Participants

Participants were 80 girls aged between 8–9 years, with a mean age of 8.43 (SD = .50) and were in Year 4 according to the U.K. National Curriculum. They were recruited from six primary schools in the southwest of England. Most of the schools were larger than the national average in size, had a lower than average number of pupils eligible for free school meals, and had a majority of White British students. The sample were all English speaking and were predominantly White (90%). Of the total participants, 47.5% of the girls were in the appearance focused-exposure condition, and 52.5% were in the non-appearance focused-exposure condition (control condition).

### Procedure

Following approval by the Research Ethics Committee at the University of the West of England and from Head teachers of all schools, a letter of introduction outlining the study and accompanying consent form was sent home to parents of all girls in Year 4. Consent forms were returned by 85 parents (overall participation rate of 28.2%) and of these, 80 girls were available to partake in the study (five girls absent on the days of the experiment).

The experimental sessions were conducted at school during normal class time and took approximately 25 min per child to complete. The study was introduced to the girls as looking at what sorts of games girls like to play and what they would like to be when they grow up. The girls were told they would be asked some questions before and after playing a game on a laptop. Before starting, girls also provided verbal assent to participate. It was emphasized that this was not a test, that there were no right or wrong answers, and the answer formats were explained carefully for each section. Each question was read aloud by the researcher, and responses were entered on a laptop.

Participants were randomly assigned to one of two experimental conditions (“appearance-focused” game and “control” game). Prior to playing the game, researchers collected data in relation to demographics and Internet use at home. Participants then played one of two games from the games site *Friv* for ten minutes, after reading the simple online instructions. In the experimental (appearance-focused) condition the girls played a game called *Dream Date Dress Up* that requires players to memorize an image of a male character’s “dream date”. Girls were then required to change the clothes and hairstyle of a female character to match this image. At the end, children received a score based on how well their final character matched the boy’s “dream date”. In the control condition the girls played a game called *Penguin Diner* that involves a penguin character working as a waiter in a café serving customers. The *Dream Date Dress Up* game was chosen as it was typical of the numerous “dress-up/make-over” games that were prevalent on the *Friv* website, and also explicitly contained the message that a girl’s value comes from her physical appearance (a key definition of sexualization, APA report). The *Penguin Diner* game was chosen as the control game as it did not contain any human figures (to elicit appearance-based comparisons), the penguins had no apparent gender, and the game appeared equally appealing and engaging to young girls.

After playing the game the game for 10 min, participants completed self-report measures of self-objectification, body dissatisfaction, and career cognitions. At the end of the study girls were thanked and given a small gift, such as a sticker or piece of stationery, for their participation.

### Measures

#### Internet use

A number of questions about Internet usage were developed. First, participants were asked whether they used the Internet at home and how often they go on the Internet each week *(Everyday*, *Most days (but not every day)*, *1*–*2 times a week*, *Hardly ever)*. Participants were then asked to indicate what they used the Internet for: *(Homework, Talking to friends (e.g., using Facebook, Kik, Instagram, Twitter or email), Watching videos (such as films, programs or YouTube), Playing games, Searching for information using Google or Bing, Sharing things (such as making YouTube videos), Other*, (more than one response option allowed), as well as to share any rules their parents set about when they could use the Internet or what they could look at whilst on the Internet. Finally, girls were asked specifically whether they played on the Internet games website *Friv* (*I never play on this site, I sometime play on this site, I play on this site a lot*).

#### Body dissatisfaction

Using the Child Figure Rating Scale (Tiggemann and Wilson-Barrett 1998) each girl was asked to point to the figure that most looks like her (current body size) and then the figure that she would most like to look like (ideal body size). Children were explicitly told that the second answer “could be the same, or it could be different” to the previous answer. A body shape dissatisfaction score was computed by subtracting the girl’s ideal from her current body size. A score of zero indicates no body shape dissatisfaction, whereas a negative score signifies a desire to be thinner, and a positive score indicates a desire to be bigger. Additionally, using the Adolescent Figure Ratings Scale (Tiggemann and Pennington 1990) each girl was asked to point to the figure that shows the way she would like to look when she is grown up (ideal adult body size). Again, girls were informed that their answer “could be the same, or it could be different” to their previous answers.

#### State self-objectification

State self-objectification was measured using the Modified Twenty Statements Test (TST) (Fredrickson et al. 1998). This measure has been used to examine differences in self-concept and to elicit descriptions of the self through free-format responses, and has been used in similar research (see Martins et al. 2007; Tiggemann and Boundy 2008). For the purpose of this study, it was shortened from ten statements to three, in line with Gapinski’s reasoning that earlier items on the Ten Statements Test would be more indicative of participants’ state self-objectification (Gapinski et al. 2003). Furthermore, using fewer items was deemed more appropriate for this age group. Participants were asked to describe themselves using three words in response to “I am…”. The original coding system used by Fredrickson et al. (Fredrickson et al. 1998) was adopted, whereby the free responses were classified into categories. For the purpose of measuring state self-objectification, we focused on the number of times that participants generated statements in the body shape and size category and physical appearance category.

#### Career cognitions and aspirations

Following Sherman and Zurbriggen (2014), girls were presented with color photographs of 11 workplaces representing different occupations or careers. One neutral, five female-dominated, and five male-dominated places of employment were pictured. Male- and female-dominated occupations were determined by data specifying the ratio of men to women employed in different occupations using UK census data, that correlates with similar data outlined by Sherman and Zurbriggen (2014) and Liben and colleagues (Liben et al. 2002). The neutral occupation was a server in a restaurant. The female-dominated occupations were: teacher, librarian, day care worker, flight attendant, and nurse. The male-dominated occupations were: construction worker, firefighter, pilot, doctor, and police officer. Each picture included a one-sentence description of the photo (e.g., “This is a restaurant: where a food server works”) and a description for the kind of work someone would do in the scene (e.g., “A food server: is someone who brings people food at a restaurant”). None of the pictures included any human figures, but held recognizable clues as to the career represented. The data collection regarding cognitions about careers was described to participants as a picture game. Originally, Sherman and Zurbriggen (2014) asked participants “Could you do this job when you grow up?” and “Could a boy do this job when he grows up?”. Here we also asked participants “Could other girls do this job when they grow up?” and “Would you like to do this job when you grow up?” The first allowed for analyses between what girls believed they could do themselves in comparison to both boys and other girls. The latter allowed for the distinction between what girls believe they could do (abilities), and what they would like to do (preferences). Participants indicated either “yes” or “no” to each question and scores were calculated by summing the number of “yes” responses.

## Results

### Internet Use

Almost all of the girls (96.3%) indicated that they used the Internet at home. Of these girls, 13.0% indicated they used the Internet every day, 33.8% used the Internet “most days”, 42.9% used the Internet 1–2 times a week, and the remaining 10.4% of girls indicated they “hardly ever” used the Internet. One third of the girls (33.3%) reported that they were allowed to use the Internet “whenever they like”, with the remainder (66.6%) reporting they could only use the Internet “with their parents’ permission”. The most common activities to do on the Internet were: playing games (reported by 81.8% of girls who used the Internet at home), searching for information (81.8%), watching videos (77.9%), doing homework (64.9%), talking to friends (23.4%), and sharing photos or videos (14.3%). When asked specifically whether they played on the games website *Friv*, 60% of girls indicated they did use this website (13.8% “a lot”, 46.3% “sometimes”).

### Similarity Between Conditions

An independent t-test confirmed no difference between the experimental and control groups for age, *t*(77) = 1.33, *p* = .18, frequency of Internet use, *t*(75) = 1.27, *p* = .21, frequency of exposure to the *Friv* website, *t*(78) = .66, *p* = .51, nor for enjoyment of the experimental and control games, *t*(78) = 1.79, *p* = .08.

### Effect of Appearance-Based Game on Body Dissatisfaction

Independent sample t-tests were conducted to assess the effect of game type on girls’ figure rating preferences (see Table 1 for means). Consistent with hypothesis, there was a significant difference in body dissatisfaction between the two conditions, *t* (78) = 2.10, *p* = .02, *d* = .46. There was no significant difference between the current figure selected by girls in the two game conditions, *t* (78) = −0.05, *p* = . 48. However, girls in the appearance game condition selected significantly thinner figures for their ideal figure, *t* (78) = −2.15, *p* = .02, *d* = −.48, and their ideal future figure, *t* (78) = −1.77, *p* = .04, *d* = −.39, compared to girls in the non-appearance game condition.

**Table 1:** Descriptive statistics for body image and career preference by condition

	Possible range	Appearance-focused game	Non appearance-focused game	*p*-value
Body image				
Body dissatisfaction (Current-Ideal)	−8 to +8	0.89	0.31	.02*
		(1.39)	(1.09)	
Current figure	1–9	4.34	4.36	.48
		(1.26)	(1.39)	
Ideal figure	1–9	3.45	4.05	.02*
		(1.31)	(1.19)	
Future ideal	1–9	3.82	4.29	.04*
		(1.27)	(1.11)	
State self-objectification	0–3	0.24	0.21	.43
		(0.63)	(0.52)	
Career preference				
Preference for female-dominated occupations	0–5	2.43	1.86	.04*
		(1.31)	(1.42)	
Preference for male-dominated occupations	0–5	1.32	1.21	.43
		(1.09)	(1.00)	

*p < .05 (one –tailed)

### Effect of Appearance-Based Game on State Self-Objectification

An independent samples t-test was conducted to assess for differences on state self-objectification by condition. There was no significant effect of condition on state self-objectification, *t* (78) = .18, *p* = .43, see Table 1.

### Effect of Appearance-Based Game on Career Potential

The means and standard deviations for the career endorsement by actor, career gender stereotype, and condition are reported in Table 2. Replicating Sherman and Zurbriggen’s (2014) analyses, a mixed design ANOVA was used to examine the impact of game type on the number of endorsements for possible occupations. A 3 actor in career (self vs. boys vs. other girls) × 2 occupation type (masculine vs. feminine) × 2 game condition (appearance vs non-appearance) ANOVA revealed a significant main effect for actor in career choice, *F*(1,78) = 73.04, *p* < .001, partial η^2^ = .48. Comparisons indicate that participants reported that boys, *F* (1, 78) = 86.28, *p* < .001, partial η^2^ = .53, and other girls, *F* (1, 78) = 75.34, *p* < .001, partial η^2^ = .49, could do significantly more jobs than they could do themselves (see Table 2). There was a significant main effect for occupation type, *F* (1, 78) = 16.43, *p* < .001, partial η^2^ = .17. However there was no significant main effect for condition, *F* (1, 78) = 1.06, *p* = .31, partial η^2^ = .01.

**Table 2:** Means (and standard deviations) for career cognitions

	Female-dominated occupations (0–5)			Male-dominated occupations (0–5)			All occupations (0–10)		
	“I can do”	“A boy can do”	“Girls can do”	“I can do”	“A boy can do”	“Girls can do”	“I can do”	“A boy can do”	“Girls can do”
Appearance-focused game	3.92	4.52	4.82	3.16	4.89	4.37	7.08	9.42	9.18
	(1.34)	(0.92)	(0.56)	(1.67)	(0.51)	(1.08)	(2.45)	(1.08)	(1.39)
Non Appearance-focused game	3.69	4.26	4.95	2.88	4.95	4.12	6.57	9.21	9.07
	(1.26)	(0.89)	(0.22)	(1.52)	(0.22)	(1.21)	(2.38)	(0.92)	(1.20)
Both games	3.80	4.39	4.89	3.01	4.93	4.24	6.81	9.31	9.13
	(1.30)	(0.91)	(0.42)	(1.59)	(0.38)	(1.15)	(2.41)	(1.00)	(1.29)

The significant effect for actor was qualified by a significant interaction between actor and occupation, *F* (2, 156) = 23.40, *p* < .001, partial η^2^ = .23. To explore this interaction, analysis was conducted separately for endorsements of feminine and masculine occupations. There was a significant effect of actor on endorsements for both feminine occupations, *F* (2, 78) = 34.75, *p* < .001, partial η^2^ = .47, and masculine occupations, *F* (2, 78) = 56.75, *p < *.001, partial η^2^ = .59. Participants reported that they could do fewer masculine jobs than boys, *t* (79) = −10.58, *p* < .001, and other girls, *t* (79) = −6.75, *p* < .001. Participants also reported that they could do fewer feminine jobs than boys, *t* (79) = −3.41, *p* < .001, and other girls, *t* (79) = −7.57, *p* < .001. Participants reported that boys could do more masculine jobs than other girls, *t* (79) = 5.33, *p < *.001. Conversely, participants reported that other girls could do more feminine jobs than boys, *t* (79) = −4.44, *p* < .001. Hence participants endorsed traditional gender expectations about career potential. In addition, participants reported that they were generally less able than both other boys and other girls of doing the occupations listed.

Critically for our hypotheses, none of the interactions involving condition were significant. Neither of the two-way interactions between actor and condition, *F* (2, 77) = .30, *p* = .74, partial η^2^ = .008, nor occupation and condition, *F* (1, 78) = .06, *p* = .80, partial η^2^ = .001, were statistically significant. Moreover, the three way interaction between actor, condition, and occupation was not significant, *F* (2, 77) = 1.52, *p* = .22, partial η^2^ = .04. Therefore, contrary to hypothesis, playing the appearance-focused game had no impact on cognitions about possible careers.

### Effect of Appearance-Based Game on Career Preferences

In addition to reporting on whether they *could* do each of the jobs listed by Sherman and Zurbriggen (2014) girls in the current study also reported whether they would *like* to do each job. Our prediction that girls would rate feminine careers more positively in the appearance-focused, compared to non-appearance, condition was supported, *t* (78) = 1.83, *p* = .04, *d* = .40, see Table 2. However, there was no significant difference in the preference for masculine careers between the two conditions, *t* (78) = .43, *p* = .67.

### Sensitivity Analysis and Power

In our analysis of career potential, we replicated Sherman and Zurbriggen (2014) and we may have been underpowered to identify interactions between game condition, actor, and occupation central to our hypotheses. Therefore, we also ran a series of t-tests comparing the “could” ratings for girls/feminine roles, girls/masculine roles, boys/feminine roles, boys/masculine roles, self/feminine roles and self/masculine roles in each condition. None of these t-test were statistically significant, all *t*s < 1.46 and all *p*s > .148.

In our other analyses, we conducted multiple t-tests to target each of our hypotheses directly. However, this increases the chance of finding significance by chance. A MANOVA across these variables confirmed a significant difference between the control and appearance-focused game group, λ = .83, *F*(5,74) = 3.04, *p* = 0.15, η_p_^2^ = .17.

## Discussion

Recent public and scholarly debate has highlighted an increase in the sexualization of girls in modern Western culture (APA 2007; Bailey 2011; Smolak and Murnen 2011). Reports such as the American Psychological Association’s (APA) Task Force on the Sexualization of Girls (2007) argue that girls are frequently exposed to messages that reinforce the importance of appearance and looking “sexy”, and that exposure to such messages may contribute to body image problems, as well as a number of other negative psychological consequences. However, there is scant existing evidence to support such claims. In recent years, the Internet has emerged as a powerful cultural influence for young people, with Internet games being a popular source of entertainment for young children (Rideout et al. 2010). The aim of the current study was to examine the impact of playing an appearance-focused Internet game on the body dissatisfaction and career cognitions and aspirations of young girls. With respect to body dissatisfaction, the results indicate that 8–9 year old girls who played an appearance-focused Internet game (*Dream Date Dress Up*) for a brief period of 10 min demonstrated heightened body dissatisfaction compared to girls who played an appearance-neutral Internet game (*Penguin Diner*). This body dissatisfaction was expressed via a preference for a thinner ideal body now as well as a preference for a thinner ideal body in the future. Our identification of a potential contributor to childhood body dissatisfaction is both important and concerning given the established links between early body dissatisfaction and later serious psychological outcomes such as dieting, disordered eating (Stice 2002), and reduced self-esteem (Wertheim et al. 2009). The findings highlight that the deleterious effects of exposure to appearance-focused and sexualized messages that have previously been demonstrated with adult women and adolescent girls (e.g., Aubrey 2006; Hargreaves and Tiggemann 2004; Harper and Tiggemann 2008; Vandenbosch and Eggermont 2012), may indeed be relevant to much younger girls.

Further, identifying contributors to body dissatisfaction in early childhood is important given the known rise in body dissatisfaction that occurs between childhood and adolescence, when a high proportion of adolescent girls express dissatisfaction with their bodies and appearance (Neumark-Sztainer et al. 2012; Ricciardelli and McCabe 2001), and the fact that early body dissatisfaction is shown to predict later body dissatisfaction (Paxton et al. 2006). While appearance-focused internet games are only one source of influence on young girls, recognizing and understanding these influences may help to curb the cumulative impact of societal influences on the development of body dissatisfaction.

The present findings add to and extend the small body of experimental work that has investigated the impact of exposure to media and toys on the body image of young girls. In the only other experimental study to investigate the impact of exposure to appearance-focused media, Hayes and Tantleff-Dunn (2010) found that exposure to film clips with appearance-related messages did *not* impact body dissatisfaction in 3–6 year old girls. The present contrasting finding, that engagement with an appearance-focused Internet game *did* impact body dissatisfaction in 8–9 year old girls, may be an indication of the different developmental stages of the two samples, with body dissatisfaction not typically established before the age of 6 (Dohnt and Tiggemann 2005). Further, it may be that Internet games are a particularly potent form of media in that they not only present appearance and body related ideals, but the interactive nature of the game typically encourages players to adopt the persona of a particular character. This adoption of a persona may aid the internalization of particular messages and ideals more so than the viewing of “traditional” media formats where one is typically viewing characters from an outside, observer’s perspective. In the *Dream Date Dress Up* game used in the present study the female character is preparing for a date with a boy. The player is shown an image of what the boy’s “dream date” looks like, and then proceeds to alter her character’s appearance (through clothes, hair styles, and accessories) in order to be appealing to the boy character. It is possible that the playing of such dress-up and make-over games encourage girls to focus on their outward physical appearance and highlights a perceived discrepancy between their own appearance and bodies and that of the culturally prescribed thin ideal. Further research to replicate and expand the current findings with young girls will be critical in order to fully appreciate the impact of engaging in this type of interactive game at a young age, as well as the longer term impact as children move into early adolescence when engagement with other life simulation video games (e.g., *The Sims*) and social networking services (e.g., *Facebook*, *Instagram*, *Snapchat*) rapidly increases (Carr 2005; Tiggemann and Slater 2013, 2014).

Two studies have examined the impact of exposure to images of Barbie and interacting with an actual Barbie doll and have found effects on either body dissatisfaction (Dittmar et al. 2006) or the internalization of a thin ideal body shape (Rice et al. 2016) in 5–8 year old girls. The degree of body dissatisfaction expressed by girls who played the appearance-focused game in the current study (on average choosing an ideal body figure that was just under 1 figure smaller than their perceived current figure) was very similar to the degree of body dissatisfaction expressed by girls exposed to images of Barbie in the Dittmar et al. (2006) study and by girls who actually played with a Barbie doll in the Rice et al. (2016) study. Perhaps exposure to toys and dolls (even when it is in print format as in the Dittmar et al. 2006 study), like the playing of particular Internet games, also encourages girls to imagine themselves as the character potentially contributing to feelings of body dissatisfaction.

Contrary to hypothesis, the present study found no evidence that exposure to an appearance-focused game impacted girls’ self-objectification. The extremely scarce existing evidence has focused on “self-sexualization” or a preference for sexualized clothing rather than using a measure of self-objectification. Slater and Tiggemann (2016) demonstrated a correlation between exposure to sexualized media (television and magazines with a focus on appearance and the body in a sexualizing way) and a preference for sexualized clothing in 6–9 year old girls, whereas Starr and Ferguson (2012) found that the quantity of media consumption (television and movie consumption) was largely unrelated to self-sexualization in girls of the same age. In contrast to these studies, the present study used a modified version of the Twenty Statements’ Test where girls were asked to describe themselves in three words as a state measure of self-objectification. There was no indication of a condition effect on this measure. However, the means were extremely low for both conditions, indicating that overall very few girls chose to describe themselves in appearance or body-related terms (e.g., “I am skinny”, “I am pretty”). To our knowledge, this measure has not been used with children of this age before, and it may be that it lacks the required sensitivity for use with this age range. Furthermore, the truncation of the measure to three statements (necessary given the age of the participants) may have also contributed to the lack of effects. Future research might usefully examine alternative measurement techniques to capture the concept of self-objectification, for example the adapted version of the Self-Objectification Questionnaire developed by Jongenelis et al. (2014) that was successfully used with girls and boys from 6 years of age, or the preference for sexualized clothing measure developed by Slater and Tiggemann (2016).

With respect to career cognitions, although there was no impact of the appearance-related game, overall girls reported that they could do significantly fewer jobs than boys could do. Sadly, these findings seemingly indicate that British girls, like their American counterparts, feel that boys can achieve more than they can when it comes to future careers. However, importantly, the current study added an additional question to the original protocol reported by Sherman and Zurbriggen (2014), and asked whether “other girls could do this job”. Interestingly this addition revealed that girls felt they could not only do significantly fewer jobs than boys, but also fewer than other girls. This suggests that the observed discrepancy between what girls themselves believe they can achieve and what boys can achieve may not necessarily be a result of the gender of the comparison target but reflects either a general modesty in self-presentation or lack of self-confidence in girls. These findings appear to align with existing research that suggests that females adopt a more modest self-presentation style than males (Zook and Russotti 2013) and that girls often display lower levels of self-confidence in their abilities and aspirations than boys (DeWitt et al. 2013).

Interestingly, although playing the appearance-focused game did not impact on what occupations girls *believed* they could do, it *did* impact on girls’ career preferences, or what occupations they *wanted* to do. Specifically, girls who played the appearance-focused game expressed a preference for traditionally feminine occupations compared to girls who played the control game. This was not the case for traditionally masculine occupations. Therefore, the appearance-focused messages inherent in the *Dream Date Dress-up* game (e.g., that girls should alter their appearance in order to be attractive to a boy) may serve to strengthen traditional gender-role stereotypes when it comes to girls thinking about their ideal future careers.

The present study was the first to investigate the impact of playing appearance-focused Internet games on young girls’ body image and career aspirations. Given the pervasiveness and the popularity of Internet-based play for young children (Rideout et al. 2010), it is critical for researchers to begin to understand the impact of engagement with this particular media format. The findings suggest that playing Internet games that emphasize the importance of appearance may not be benign for young girls. Although further research will be necessary to confirm and further elucidate these findings, at this stage they suggest a number of practical implications. For parents and educators, being aware of the games that young girls are playing, and ideally limiting exposure to those that are laden with appearance messages, appears prudent. This may be difficult in a societal landscape that is increasingly focused on appearance and brimming with products and media heavy with sexualized messages that are strategically marketed at an increasingly young audience (Graff et al. 2013; Murnen et al. 2016). For game makers and marketers, a challenge appears to be to produce and promote games that appeal to girls without reinforcing gender role stereotypes and narrow ideals of feminine beauty. Policy makers could be encouraged to consider ways of reducing or eliminating such products.

Although the current research presents some novel and interesting findings, they must be considered in light of the study’s limitations. First, the impact of short-term exposure to only one particular game was explored. However, given that this style of game (dressing-up, make-over) is extremely common and popular with girls of this age (Kafai 1996; Subrahmanyam and Greenfield 1998; Willett 2008), the findings are noteworthy. Future research could usefully attempt to measure whether the game characters were relatable and indeed whether girls are aware of any social comparison processes. Second, the sample is homogenous in terms of ethnicity and socioeconomic background, and findings may not be generalizable. Finally, the current study examined the impact of Internet games on girls, and future research will ideally expand to examine the impact of different types of Internet play on young boys.

## Conclusion

The present study has made an important first step in investigating the impact of appearance-focused Internet games on the body image and career aspirations of young girls. Girls who played an appearance-focused Internet game for 10 min expressed greater body dissatisfaction compared to girls who played an appearance-neutral game. In addition, the appearance-focused game contributed to an increased preference for traditionally feminine occupations. The study illuminates that exposure to particular types of Internet games may be detrimental to girls, not only in terms of how they come to view their bodies, but also in terms of their future aspirations, and that these effects may be apparent at a much earlier age than previously thought. Internet games should be included in our consideration of influential sources of appearance-based messages for young girls.
